# p75 neurotrophin receptor regulates craniofacial growth and morphology in postnatal development

**DOI:** 10.3389/fcell.2025.1569533

**Published:** 2025-03-18

**Authors:** Byron Zhao, Jinsook Suh, Yan Zhang, Eric Yin, Chiho Kadota-Watanabe, In Won Chang, Jun Yaung, Isabelle Lao-Ngo, Nathan M. Young, Reuben H. Kim, Ophir D. Klein, Christine Hong

**Affiliations:** ^1^ Division of Orthodontics, Department of Orofacial Sciences, University of California, San Francisco, San Francisco, CA, United States; ^2^ Division of Maxillofacial and Neck Reconstruction, Department of Maxillofacial Orthognathics, Institute of Science Tokyo, Tokyo, Japan; ^3^ Shapiro Family Laboratory of Viral Oncology and Aging Research, School of Dentistry, University of California, Los Angeles, Los Angeles, CA, United States; ^4^ Department of Orthopaedic Surgery, University of California, San Francisco, San Francisco, CA, United States; ^5^ Department of Orofacial Sciences, Institute for Human Genetics, University of California, San Francisco, San Francisco, CA, United States; ^6^ Department of Pediatrics, Cedars-Sinai Guerin Children’s, Los Angeles, CA, United States

**Keywords:** p75^NTR^, CD271, NGF, craniofacial development, craniofacial morphogenesis, calvarial development, geometric morphometric analysis

## Abstract

Craniofacial abnormalities are among the most prevalent congenital defects, significantly affecting appearance, function, and quality of life. While the role of genetic mutations in craniofacial malformations is recognized, the underlying molecular mechanisms remain poorly understood. In this study, we investigate the role of p75 neurotrophin receptor (p75^NTR^) in craniofacial development by comparing wild-type (p75^NTR+/+^) mice against p75^NTR^-deficient (p75^NTR−/−^) knockout mice. We employed histology, micro-CT surface distance, volumetric analysis, and geometric morphometric analysis to assess craniofacial development and growth. On postnatal day 7 (P7), p75^NTR−/−^ mice exhibited reduced skull length compared to wild-type controls. By P28, micro-CT analysis revealed significant reductions in calvarial bone volume and trabecular bone thickness in p75^NTR−/−^ mice. Geometric morphometric analysis identified significant shape alterations in the nasal, parietal, and occipital regions, with p75^NTR−/−^ mice showing a shortened cranium and tapered nasal bone morphology. These findings highlight the critical role of p75^NTR^ in regulating postnatal craniofacial development. Disruption of p75^NTR^ signaling impairs both the growth and morphological integrity of craniofacial structures, which may contribute to the pathogenesis of congenital craniofacial abnormalities. In the future, a better understanding of the molecular mechanisms through which p75^NTR^ mediates craniofacial development may offer valuable insights for future targeted therapeutic strategies for craniofacial defects.

## 1 Introduction

Craniofacial abnormalities and malformations are a group of congenital anomalies that represent a third of all birth defects and affect around 1 in every 100 newborns, significantly impacting an individual’s appearance, speech, mastication, and occlusion (Corsello and Giuffrè, 2012; Twigg and Wilkie, 2015; Ueharu and Mishina, 2023). Despite their high prevalence and substantial clinical burden, the factors driving craniofacial development and the etiology of craniofacial abnormalities remain poorly understood. These conditions frequently necessitate multidisciplinary interventions and invasive surgeries that reduce the quality of life without addressing the underlying molecular causes (Neben et al., 2016; Opriş et al., 2022; Manlove et al., 2020; Shaw, 2004).

Skeletal dysplasias are a group of genetic disorders that disrupt the development, growth, and homeostasis of bones and cartilage, constituting a significant subset of congenital disorders. With over 450 recognized conditions, these disorders vary widely in severity, from mild abnormalities to life-threatening complications (Neben et al., 2016; Warman et al., 2011). Craniofacial defects associated with skeletal dysplasia arise from complex interactions among transcription factors, growth factors, and receptors, which orchestrate the genetic patterning and morphogenesis of craniofacial structures (Neben et al., 2016; Neben and Merrill, 2015).

Craniofacial structures predominantly originate from cranial neural crest cells, which differentiate into various cell types including osteoblast, chondrocytes, adipocytes, melanocytes, neural cells, and others (Bhatt et al., 2013; Achilleos and Trainor, 2012). Multiple signaling pathways are critical regulators of cranial neural crest cells (Pereur and Dambroise, 2024; Nuckolls et al., 1999; Xu et al., 2023; Sun et al., 2024). Among these, the fibroblast growth factor (FGF) signaling pathway is a key regulator of craniofacial development and calvarial bone formation. FGF signaling orchestrates epithelial-mesenchymal interactions essential for the development of various craniofacial structures, (Prochazkova et al., 2018; Teshima et al., 2016; Klein et al., 2008). Its functions are highly tissue-specific, utilizing diverse mechanisms to regulate development.

The FGF pathway also has a well characterized role in calvarial bone, regulating osteogenesis, chondrogenic proliferation and maintaining sutures, with dysregulation of FGF receptors 1-3 (*FGFR1*, *FGFR2*, *FGFR3*) being known to cause calvarial abnormalities in murine models (Neben et al., 2016; Rice et al., 2000; Richbourg et al., 2024; Marcucio et al., 2019). Dysregulation of FGFR3, which reduces chondrocyte proliferation, is associated with conditions such as achondroplasia and thanatophoric dysplasia, both characterized by distinct craniofacial anomalies (Neben et al., 2016; Rousseau et al., 1994; Shiang et al., 1994; Henderson et al., 2000). Molecular studies have demonstrated that the severity of these skeletal dysplasias correlates with the degree of FGFR3 signaling activation through the MAPK or STAT1 pathways (Laederich and Horton, 2010; Krejci et al., 2008). Consequently, inhibiting these over-activated pathways with statins has shown promise as a therapeutic strategy, as statin treatment successfully corrected bone development abnormalities in mouse models (Yamashita et al., 2014). These results highlight the critical role of mouse models in driving progress toward effective therapies for craniofacial disorders linked to congenital skeletal abnormalities.

Signaling pathways are also able to crosstalk with one another to influence craniofacial development. Sonic hedgehog (SHH) signaling pathway has a characterized function regulating mitotic activity and spatial organization within midfacial growth zones (Young et al., 2010). However, when the SHH and FGF pathways interact together they gain new functions, becoming capable of regulating the migration, survival and maintenance of neural crest progenitors (Prochazka et al., 2015; da Costa et al., 2018). Another example of pathway crosstalk involves the bone morphogenetic protein (BMP) signaling pathway, which often functions in opposition to FGF signaling. BMP signaling is known to attenuate FGF signaling in calvarial development (Ueharu and Mishina, 2023; Maruyama et al., 2010). Further studies have shown that interplay between the BMP and FGF signaling pathways help regulate calvarial bone development and injury repair, emphasizing the complex nature of calvarial development (Maruyama et al., 2010; Maruyama et al., 2017).

BMP signaling is also known for its role in regulating proliferation, differentiation, apoptosis, and migration of cranial neural crest cells. Defects in BMP signaling have been linked to craniofacial skeletal deformities including craniosynostosis in murine models, emphasizing its role in calvarial bone development (Ye et al., 2022; Mimura et al., 2016; Wang et al., 2024; Ueharu and Mishina, 2023). Specifically, BMP4 plays a significant role in calvarial sutures, where it has been shown to induce expression of *Msx* genes, key regulators of osteogenesis essential for bone formation and development (Kim et al., 1998). Interestingly, reduced BMP4 activity has been associated with increased expression of the p75 neurotrophin receptor (p75^NTR^), suggesting a functional relationship between BMP4 signaling and p75^NTR^ in the calvaria, presenting a novel area for further research (Mimura et al., 2016).

p75^NTR^ is a membrane spanning protein in the tumor necrosis factor receptor family that can bind any neurotrophin (NGF, BDNF, NT-3, NT-4) with low-affinity (Meeker and Williams, 2015). Among these potential ligands, p75^NTR^ is best known for its role as an NGF receptor (Liu et al., 2022). As a neurotrophin receptor, p75^NTR^ has a well characterized role in the nervous system mediating neuronal cell survival, regulating synaptic transmission/axial elongation, and acting as a potential marker gene for various neurodegenerative diseases such as Alzheimer’s, schizophrenia, and dementia (Jourdi et al., 2024; Dechant and Barde, 2002; Liu et al., 2022; Beattie et al., 2002).

Interestingly, p75^NTR^ (also known as CD271) is widely expressed on the surfaces of migrating neural crest cells throughout their development (Tomellini et al., 2014). It has been established as one of the most reliable surface markers to isolate neural crest cell progenitors and bone marrow stem cells (Álvarez-Viejo et al., 2015; Bühring et al., 2007; Quirici et al., 2002; Tomellini et al., 2014). In our study utilizing three distinct sets of surface markers (CD51/CD140a, CD271, and STRO-1/CD146) to isolate putative stem cell populations from primary craniofacial tissue cultures, CD271 was merged as the most effective single marker for identifying progenitor populations (Alvarez et al., 2015a).

Emerging evidence highlights the critical role of p75^NTR^ in bone development. Our previous studies demonstrated that p75^NTR^ positive mesenchymal stem cells (MSCs) from various craniofacial tissues exhibit the highest osteogenic potential with strong upregulation of key osteogenic markers such as DLX5, RUNX2, and BGLAP (Alvarez et al., 2015a; Alvarez et al., 2015b). Additionally, our lab has further characterized the role of p75^NTR^ in the osteogenesis of craniofacial MSCs both *in vitro* and *in vivo*, revealing that *Dlx5*, a master osteogenic gene, is epigenetically activated by Lysine Demethylase 4B (KDM4B) *via* p75^NTR^-mediated NGF signaling (Liu et al., 2022). These findings were confirmed *in vivo* using calvarial defect regeneration mouse model. (Liu et al., 2022). Recent studies by Wang et al. also showed that p75^NTR^ knockout mice have reduced alveolar bone mass and less osteogenic differentiation of ectomesenchymal stem cells (Wang et al., 2020).

Despite these breakthroughs, the role of p75^NTR^ in craniofacial bone development remains uncharacterized. In our study, we aimed to elucidate the impact p75^NTR^ has on craniofacial growth and morphogenesis by deleting *p75* ^*NTR*^ in mice and comparing wild-type (p75^NTR+/+^) and p75^NTR^-deficient (p75^NTR−/−^) knockout mice. Using whole-mount skeletal staining and micro-CT imaging, we assessed skull length, bone volume, bone density, and the microarchitecture of the craniofacial skeleton. Geometric morphometric analysis then allowed us to examine shape differences in the calvaria, comparing p75^NTR+/+^ and p75^NTR−/−^ mice. Our findings revealed that p75^NTR^ is critical for calvarial integrity during postnatal development, providing insight into the underlying mechanism behind craniofacial morphogenesis and opening potential pathways towards addressing craniofacial abnormalities in the future.

## 2 Results

### 2.1 p75^NTR^-deficiency leads to generalized reduction in postnatal craniofacial bone formation

**FIGURE 1:**
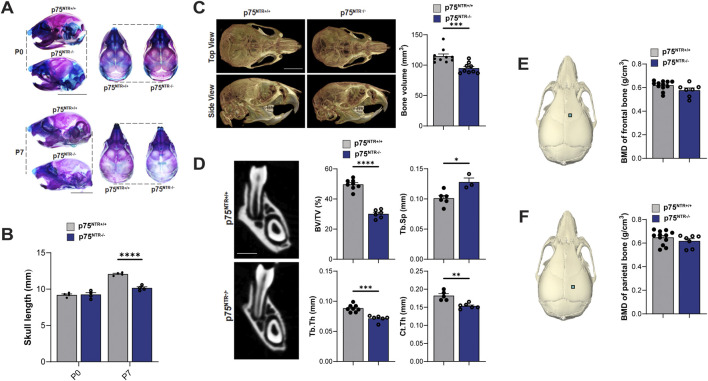
p75^NTR^ deficiency leads to a generalized reduction in postnatal craniofacial bone formation. **(A)** Whole-mount skeletal staining of skulls with alcian blue and alizarin red S. **(B)** Comparison of skull length at P0 and P7 are plotted. n = 4 **(C)** total skull volume at P28 is illustrated and plotted. n = 9–10 **(D)** Mandibular bone volume fraction (BV/TV), trabecular bone thickness (Tb.Th.) and, cortical bone thickness (Cb.Th.), and trabecular spacing (Tb.Sp.) are illustrated and plotted. n = 5–8 **(E, F)** ROI for bone mineral density (BMD) analysis is indicated with a square on the frontal and parietal bones. BMD values are plotted. n = 7–12 Bar graphs show mean ± standard deviation. Statistical significances are indicated, n.s non-significant, *p ≤ 0.05, **p ≤ 0.01, ***p ≤ 0.001, ****p ≤ 0.0001.

Beyond its well-established role in neuronal survival, p75^NTR^ has emerged as a potential key regulator of osteogenic differentiation and bone development, prompting us to examine how p75^NTR^ deficiencies may influence postnatal craniofacial bone development. p75^NTR−/−^ mice were born without complications and had no statistically significant differences in size weight, or growth compared to p75^NTR+/+^ littermates at P0. Whole-mount skeletal staining revealed no craniofacial growth defects in neonatal p75^NTR−/−^ mice at P0 (Figures 1A, B). However, as early as 7 days after birth (P7), whole-mount skeletal staining demonstrated a notably reduced skull length in p75^NTR−/−^ mice compared with p75^NTR+/+^ mice. (Figures 1A, B). Similarly, micro-CT analysis of P28 animals revealed a significant decrease in calvarial bone volume in p75^NTR−/−^ mice compared to p75^NTR+/+^ littermates (Figure 1C). Further analysis of trabecular bone microarchitecture in the region below the mandibular first molars demonstrated that the loss of p75^NTR^ resulted in decrease in bone volume in p75^NTR−/−^ mice compared to p75^NTR+/+^ mice at P28 with significantly reduced thickness of trabecular bone (Tb.Th.) and cortical bone (Cb.Th.), and increased trabecular spacing (Tb.Sp.) in p75^NTR−/−^ mice compared to p75^NTR+/+^ mice (Figure 1D). Although calvarial bone mineral density (BMD) values of the frontal bone and parietal bone (Figures 1E, F) were lower in p75^NTR−/−^ mice, the differences were not statistically significant. Collectively, these results suggest that p75^NTR^ plays a critical role in postnatal craniofacial bone development and growth.

### 2.2 p75^NTR^-deficiency leads to generalized reduction in 4-week-old craniofacial bone size

**FIGURE 2:**
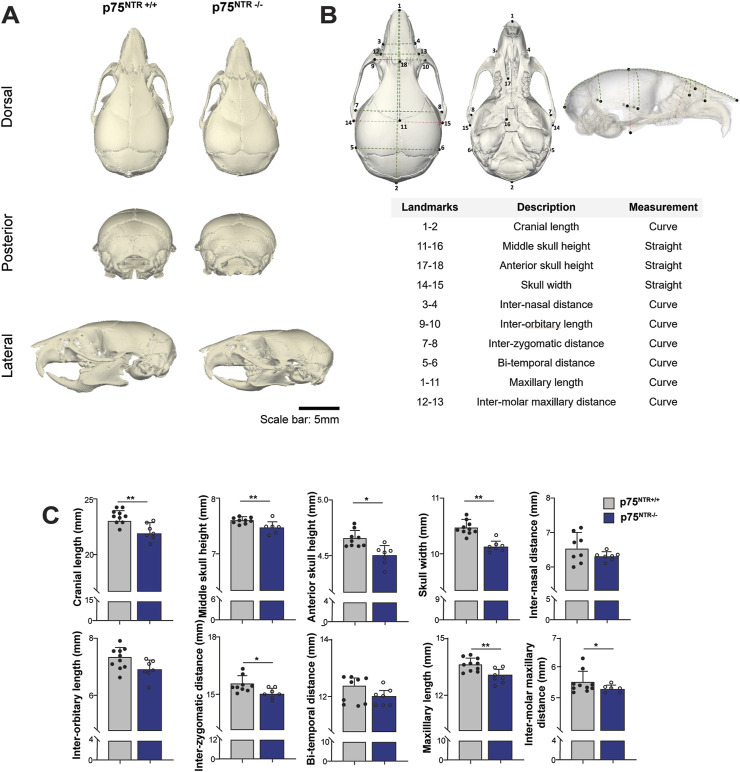
Length analysis shows cranium size reduction due to p75^NTR^ deficiency. **(A)** Representative skull rendering for visual comparison. **(B)** 18 landmarks were placed on the cranium and used to access the surface and linear distances of 10 measurements. **(C)** Comparison of distances are illustrated. n = 7–10. Bar graphs show mean ± standard deviation. Statistical significances are indicated, *p ≤ 0.05, **p ≤ 0.01.

Side-by-side visual comparison of p75^NTR+/+^ and p75^NTR−/−^ P28 mouse skulls revealed a generalized reduction in calvarial skeletal dimensions in all three views in p75^NTR−/−^ mice (Figure 2A). To further analyze the differences, we performed distance analysis using landmarks indicated in Figure 2B. These landmarks are selected to represent the overall morphology of the skull. Furthermore, these landmarks are located at transition points or boundaries between different bones so that they could be registered with high reproducibility (Kawakami and Yamamura, 2008; Liang et al., 2023). The analysis measured ten 3-dimensional (3D) surface and linear distances in the calvaria, providing detailed insights into the observed differences (Figure 2B). All ten measurements were reduced in p75^NTR−/−^ mice compared to p75^NTR+/+^ mice with seven measurements showing statistically significant differences, including cranial length, skull width, middle skull height, anterior skull height, maxillary length, inter-zygomatic distance, and inter-molar maxillary distance. These dimensions were significantly smaller in p75^NTR−/−^ mice compared to p75^NTR+/+^ littermates (Figure 2C). In contrast, decreases in inter-orbitary, inter-nasal, and bi-temporal surface distances were not statistically significant.

**FIGURE 3:**
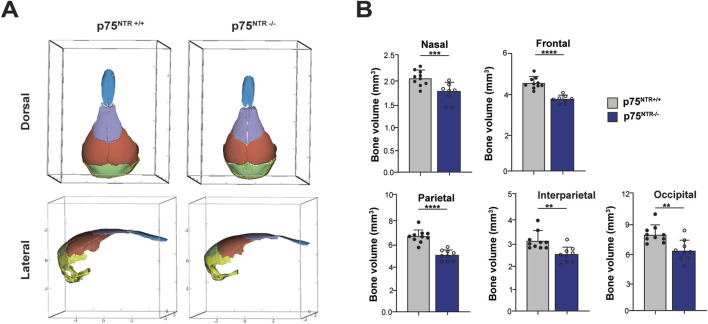
Volumetric analysis shows cranium volumetric reduction due to p75^NTR^ deficiency. **(A)** three-dimensional skull bone segmentation of nasal (blue), frontal (purple), parietal (orange), interparietal (green), and occipital (yellow). The three-dimensional coordinate boxes are in units of millimeters (mm). **(B)** bone segment volume and statistical significance in length difference is illustrated. n = 8–10. Bar graphs show mean ± standard deviation. Statistical significances are indicated, *p ≤ 0.05, **p ≤ 0.01, ***p ≤ 0.001, ****p ≤ 0.0001.

Next, we performed 3D segmentation and volumetric analysis of calvarial bones. This analysis revealed a significant 15%–20% decrease in the volumes of nasal, frontal, interparietal, parietal, and occipital bones (Figures 3A, B). In conclusion, these findings demonstrate a significant overall and specific volumetric reduction in p75^NTR−/−^ mice, suggesting that p75^NTR^ has a direct role in the developmental growth of the craniofacial bones.

### 2.3 p75^NTR^-deficient mice exhibit altered cranium morphology

While size and volume offer valuable quantitative insights into craniofacial growth, morphology is critical for capturing the intricacies and complexities of craniofacial development (Hassan et al., 2020; Young et al., 2016). Notably, size reductions were not uniformly observed across all distance measurements or volume data (Figures 2, 3), necessitating us to further investigate the role of p75^NTR^ in craniofacial morphogenesis.

**FIGURE 4:**
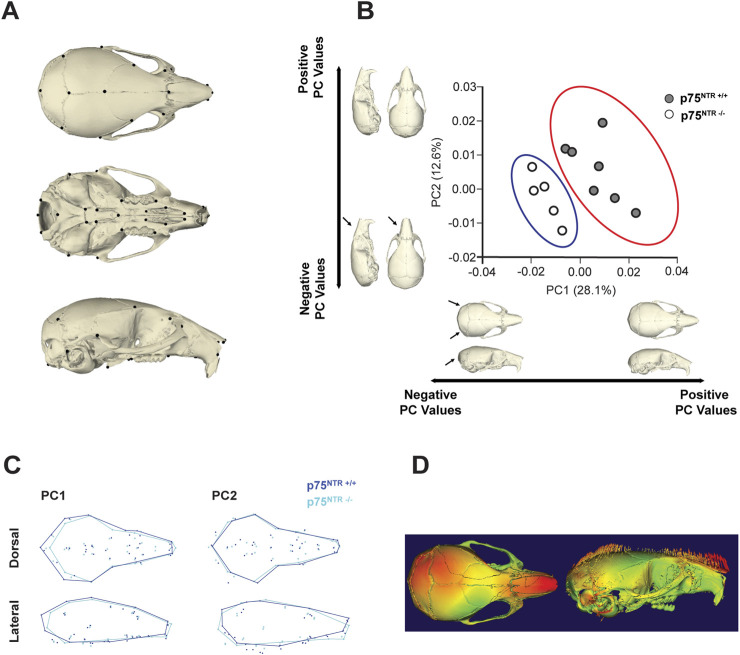
GM analysis shows morphological change due to p75^NTR^ deficiency. **(A)** Skeletal Landmarks used for GM analysis. **(B)** Scatter plots by the two first Principal Components show differences in shape between the p75^NTR+/+^ (light blue dots) and p75^NTR−/−^ (orange dots) P28 mice cranium. Black arrows indicate anatomical positions with the most prominent morphological differences between the p75^NTR+/+^ and p75^NTR−/−^ P28 mice cranium. Light blue and orange shaded ellipses are the 95% confidence ellipses for p75^NTR+/+^ (light blue ellipse) and p75^NTR−/−^ (orange ellipse) P28 mice cranium. **(C)** Wireframes showing superimposition of p75^NTR+/+^ (blue) and p75^NTR−/−^ (cyan) 4-week mice cranium demonstrate the shape changes that are associated with PC 1 and PC 2. Wireframes were generated from landmarks indicated in geometric morphometric analysis to provide a visual representation and demonstrate shape differences between p75^NTR+/+^ and p75^NTR−/−^ groups**.** Based on wireframe analysis, p75^NTR−/−^ mice exhibited reduced occipital bone size and demonstrated more angular and tapered nasal bone morphology. **(D)** The displacement heatmap (left) shows morphological differences distributed in the nasal, parietal, and inter-parietal bone regions. Arrows (right) indicate the direction of change, representing the difference between p75^NTR+/+^ and p75^NTR−/−^ samples. n = 5–7.

Here, we utilized the geometric morphometric analysis technique to compare and quantify the calvarial shapes of p75^NTR+/+^ and p75^NTR−/−^ mice in a precise approach. 41 landmarks were placed on the cranium for geometric morphometric analysis (Figure 4A; Table 1) (Shen et al., 2013; Richtsmeier et al., 2000; Hill et al., 2009; Willmore et al., 2009). Scatter plots of PC 1 and PC 2 showed distinct clusters for the p75^NTR+/+^ and p75^NTR−/−^ mouse crania (Figure 4B). For cranium, we observed a clear separation of the 95%-confidence-ellipses along the PC 1 axis, accounting for 28.1% of the total variance. The Procrustes MANOVA conducted on shape data and centroid size data demonstrates significant differences between p75^NTR+/+^ and p75^NTR−/−^ mouse crania (p < 0.0001).

**TABLE 1:** Name and descriptions for all 41 landmarks used in geometric morphometric analysis.

#	Name	Landmark description
1	iflac	Intersection of frontal process of maxilla with frontal and lacrimal bones, left side
2	rflac	Intersection of frontal process of maxilla with frontal and lacrimal bones, right side
3	lpto	Intersection of parietal, temporal and interparietal bones, left side
4	rpto	Intersection of parietal, temporal and interparietal bones, right side
5	pari	Intersection of parietal bones with anterior aspect of interparietal bone at midline
6	nas	Nasion: intersection of nasal bones, caudal point
7	nsl	Nasale: end of intersection line of nasal bones, rostral point
8	pns	Posterior nasal spine, most posterior projection of the posterior nasal spine
9	lptyp	Most inferior aspect of posterior tip of medial pterygoid process, left side
10	rptyp	Most inferior aspect of posterior tip of medial pterygoid process, right side
11	opi	Opisthion, midsagittal point on the posterior margin of the foramen magnum
12	laalf	Most anterior point of the anterior palatine foramen, left side
13	raalf	Most anterior point of the anterior palatine foramen, right side
14	lfsq	Frontal-squasmosal intersection at temporal crest, left side
15	rfsq	Frontal-squasmosal intersection at temporal crest, right side
16	lmax	Center of alveolar ridge over maxillary incisor, left side
17	rmax	Center of alveolar ridge over maxillary incisor, right side
18	lmxph	Lateral intersection of maxilla and palatine bone posterior to the third molar, left side
19	rmxph	Lateral intersection of maxilla and palatine bone posterior to the third molar, right side
20	lorb	Anterior notch on frontal process lateral to infraorbital fissure, left side
21	rorb	Anterior notch on frontal process lateral to infraorbital fissure, right side
22	lpalf	Most posterior point of the anterior palatine foramen, left side
23	rpalf	Most posterior point of the anterior palatine foramen, right side
24	lmaxna	Anterior-most point at intersection of premaxillae and nasal bones, left side
25	rmaxna	Anterior-most point at intersection of premaxillae and nasal bones, right side
26	lpmx	Most inferior lateral point on premaxilla-maxilla suture, left side
27	rpmx	Most inferior lateral point on premaxilla-maxilla suture, right side
28	lsqzy	Joining of squamosal body to zygomatic process of squamosal, left side
29	rsqzy	Joining of squamosal body to zygomatic process of squamosal, right side
30	bas	Basion, midsagittal point on the anterior margin of the foramen magnum
31	brg	Bregma: intersection of frontal bones and parietal bones at midline
32	paro	Intersection of interparietal bones with squamous portion of occipital bone at midline
33	leam	Most posteroinferior point on the superior portion of the tympanic ring, left side
34	ream	Most posteroinferior point on the superior portion of the tympanic ring, right side
35	lama	The anterior most point on the central ant/post axis of the left molar alveolus
36	rama	The anterior most point on the central ant/post axis of the right molar alveolus
37	lcc	Most anterior medial point on the left carotid canal
38	rcc	Most anterior medial point on the right carotid canal
39	ans	Anterior nasal spine, most anterior point of inter-premaxillary suture at base of nasal aperture
40	rfmc	Intersection of the right occipital condyle and the foramen magnum, taken at the lateral most curvature, right side
41	lfmc	Intersection of the right occipital condyle and the foramen magnum, taken at the lateral most curvature, left side

Wireframe and displacement heatmap reveal morphological differences primarily distributed across the nasal, parietal, and interparietal bone regions (Figures 4C, D). In particular, PC1 indicates that the most significant shape variation is located in the caudal region of the cranium. In p75^NTR−/−^ mice, the cranium is shorter overall, driven by shape differences associated with the shortening and narrowing of the occipital and interparietal bones. Additionally, PC2 highlights a ventral bending of the nasal-palatal portion of the cranium in p75^NTR−/−^ mice compared to p75^NTR+/+^ mice, despite no apparent narrowing or shortening of the nasal bones.

In summary, geometric morphometric analysis demonstrates that p75^NTR^ deficiency led to significant overall morphological alterations. These findings underscore the direct involvement of p75^NTR^ in cranial morphogenesis.

## 3 Discussion

Our findings highlight the critical role of p75^NTR^ in craniofacial bone development, growth, and morphogenesis. By integrating histology, surface distance and linear length analysis, 3D segmentation, volumetric measurements, and geometric morphometric analysis, we demonstrated how p75^NTR^ deficiency leads to significant postnatal deficits in craniofacial development. Although p75^NTR^-deficient mice were born without notable differences in size, weight, or craniofacial structure compared to p75^NTR+/+^ littermates, abnormalities became evident shortly after birth, emphasizing its essential role in postnatal growth and morphogenesis rather than embryonic development.

Craniofacial development is a uniquely complex process driven by region-specific and mosaic growth patterns requiring precise spatial and temporal coordination (Feng et al., 2009; Roth et al., 2021). Unlike the predominantly linear growth of long bones, craniofacial structures develop through intricate, shape-dependent mechanisms (Breur et al., 1991; Geetha-Loganathan et al., 2022; Kronenberg, 2003). Variations in these processes are evident in craniofacial syndromes, where growth disruptions affect multiple dimensions of the skull, underscoring the critical role of shape in both function and aesthetics (Bartzela et al., 2017; Geetha-Loganathan et al., 2022). Consequently, advanced shape analysis is essential for understanding these mechanisms and the contributions of p75^NTR^ to craniofacial development (Matthews et al., 2022).

In this study, geometric morphometric analysis proved invaluable for quantifying craniofacial shape changes caused by p75^NTR^ loss-of-function. Unlike traditional morphometric methods focused solely on size, geometric morphometric analysis captures subtle, localized shape variations by analyzing spatial relationships between craniofacial landmarks (Agbolade et al., 2020; Rutland et al., 2021; Hallgrimsson et al., 2015; Hassan et al., 2020; Young et al., 2016). Our findings revealed significant alterations in the nasal, interparietal, and occipital regions of p75^NTR^-deficient mice, highlighting the role of p75-mediated signaling in craniofacial symmetry and proportionality. Furthermore, the clustering along principal component axes demonstrated clear differences in overall craniofacial shape between p75^NTR+/+^ and p75^NTR−/−^ mice. Together with volumetric and length analysis, our results suggest that p75^NTR^ plays an essential role not only in overall bone size but also in cranial shape and structural integrity.

The C57BL/6J mouse used in this study exhibits transverse and vertical growth of its craniofacial skeleton until 4 weeks of age (Vora et al., 2015). Therefore, studying the p75^NTR^’s involvement in craniofacial development at 4 weeks (P28) provides insights into its function during early postnatal development. While significant craniofacial defects were observed during this period, the long-term effects of p75^NTR^ deficiency remain unclear. Future studies should examine older mice, such as 3-month-olds and 12-month-olds, after growth completion to determine whether craniofacial defects persist or worsen over time or whether compensatory mechanisms mitigate their effects.

Another avenue for future research is using inducible tissue-specific conditional knockout mice rather than whole-body knockouts to better delineate the role of p75^NTR^ in craniofacial development (Hall et al., 2009). This approach allows for targeted deletion of p75^NTR^ in specific cell types, such as osteoblasts or craniofacial progenitor cells, at controlled developmental stages. This strategy would minimize systemic confounding factors by isolating tissue-specific effects and offer more precise insights into how p75^NTR^ regulates craniofacial growth and form. Furthermore, while our study focused on genotype-dependent effects, the sex-specific differences in skeletal development may contribute to craniofacial variation in p75^NTR^ deficient animals. Future studies should include sex-stratified analyses to further elucidate potential interactions between genetic factors and sex-specific growth patterns.

Our previous studies have demonstrated that NGF promotes osteogenic differentiation of human craniofacial MSCs *via* p75^NTR^ (Liu et al., 2022). This process involves downstream activation of the JNK cascade and the identification of KDM4B as a key epigenetic regulator of NGF-p75-mediated osteogenesis (Liu et al., 2022). However, the specific ligands and signaling pathways regulating craniofacial bone development through p75^NTR^ remain to be fully elucidated. Identifying such ligands, particularly NGF, and further characterizing their molecular interactions would provide critical insights into the mechanisms underlying craniofacial development and set the stage for therapeutic applications.

Furthermore, histological and molecular analyses are needed to elucidate the specific stages of bone formation regulated by p75^NTR^. Investigating gene and protein expression markers such as Runx2, Sox9, and Col2a1 could provide deeper insights into the molecular mechanisms involved. Future research combining geometric morphometric analysis, volumetric analysis, histology, and molecular profiling across different craniofacial and endochondral bone tissues will enhance our understanding of p75^NTR^’s role in skeletal development.

From a clinical perspective, a deeper understanding of p75^NTR^’s role in craniofacial development has the potential to lead to impactful therapeutic applications. BMP4, a key regulator of cranial neural crest cells and osteogenesis, appears to influence p75^NTR^ expression (Mimura et al., 2016). BMP2, a closely related protein, is already used clinically to promote bone remodeling and is a viable alternative to bone grafts (Halloran et al., 2020). This suggests that targeting BMP4 or downstream p75^NTR^-mediated signaling could yield new therapeutic strategies. However, further research is necessary to realize these clinical possibilities fully.

In conclusion, p75^NTR^ deficiency leads to significant morphological alterations and reductions in craniofacial bone size, highlighting the critical role of p75^NTR^ in craniofacial development. The combined use of geometric morphometric and volumetric analyses proved to be a robust approach for functional genetic studies and developmental research. These methods complement each other, with geometric morphometric analysis providing insights into overall shape variation and volumetric analysis supplementing size-related information. This integrative phenotypic analysis serves as a strong foundation for future studies to elucidate p75^NTR^-related pathways in craniofacial dysmorphism and developmental delays, with potential applications in related research fields.

## 4 Materials and methods

### 4.1 Animals

p75^NTR−/−^ and p75^NTR+/+^ mice were purchased from the Jackson Laboratory (Bar Harbor, ME) and housed inside the Laboratory Animal Resource Center facilities at the University of California, San Francisco (UCSF). The Animal Research Committee (ARC) at UCSF approved and regulated all protocols used in animal experiments. The mice were bred to produce heterozygous females and males, which were then used to produce mice with different genotypes for this study. Both sexes were included for all analyses.

### 4.2 Whole-mount skeletal staining

The skin and internal organs of P0 and P7 mice were removed for morphological analysis. After overnight fixation in 95% ethanol at room temperature, the mice were stained in Alcian blue solution (0.03% Alcian blue in 20% acetic acid/ethanol) overnight. After several hours in 70% and 95% ethanol, they were transferred to a 1% potassium hydroxide (KOH) solution for 2 h to digest and clear the tissue. After staining in Alizarin red S solution for 4 h (0.005% Alizarin red in 1% KOH), skeletons were cleared in a series of 1% KOH/25% glycerol, 0.5% KOH/50% glycerol and stored in 0.5% KOH/70% glycerol at 4°C. Images of the stained skeletons and cartilages were taken under a stereomicroscope (MVX10; Olympus, Tokyo, Japan).

### 4.3 Micro-computed tomography (Micro-CT) analysis for bone tissues

After 4% paraformaldehyde fixation overnight, P28 skulls of the mice were subjected to micro-CT scanning. Microarchitectures of the cortical bone of the skulls were measured by Skyscan 1,275 μCT (Bruker, Kontich, Belgium) as previously described (Hsu et al., 2016). Bones were placed vertically in a scanning holder and scanned at the following settings: 10 μm resolution, 60 kVp energy, 166 μA intensity, 0.3-degree rotation, and an integration time of 200 ms. Two-dimensional (2D) slices from each skull were combined using NRecon and CTAn/CTVol programs (Bruker) to form a 3D reconstruction and quantify BV/TV (%), Tb.N. (mm-1), Tb.Th. (mm), and Tb.Sp. (mm) to determine the trabecular bone microarchitecture in the region below the mandibular first molar. To analyze bone mineral density (BMD), three regions of interest (ROI) on craniofacial bone tissues were used as previously described (Wei et al., 2017). All ROIs were set to 0.4 mm × 0.4 mm in size (Figures 1E,F). The first ROI was set on the frontal bone, which is located 1.5 mm anterior to the intersection point of the coronal suture and sagittal suture and 1 mm lateral to the posterior frontal suture. The second area to measure BMD of parietal bone was located 1.5 mm posterior to the intersection point of the coronal suture and sagittal suture and 1.5 mm lateral to the sagittal suture. The thickness of the ROIs was 0.4 mm to generate BMD by using 40 slices from posterior to the initial plane.

### 4.4 Surface-length and straight-line-length analysis

We imported Micro-CT data into 3D Slicer software (slicer.org, Massachusetts, United States) and manually segmented the cranium bone. We utilized a constant rendering threshold of 1471 HU to 9800 HU to generate isosurfaces for each anatomical region. We then placed 18 landmarks to encapsulate the cranium morphology. Using these landmarks, we accessed surface-outline distances and straight-line distances. Surface-outline spans across the skull and extrapolates the 3D size information; in comparison, the straight-line distances analyze the distance between two landmarks in a 2D manner. Seven 4-week-old p75^NTR+/+^ mice and five 4-week-old p75^NTR−/−^ mice were considered for the evaluation.

### 4.5 Volumetric analysis

We imported Micro-CT data into 3D Slicer software (slicer.org, Massachusetts, United States), and we manually segmented the nasal, interparietal, parietal, occipital, and frontal bones of the skull. We utilized a constant rendering threshold of 1471 HU to 9800 HU to generate isosurfaces for each anatomical region. Ten 4-week-old p75^NTR+/+^ mice and nine 4-week-old p75^NTR−/−^ mice were considered for the evaluation. We then calculated the volume of each bone segment and evaluated the statistical difference between the p75^NTR+/+^ and p75^NTR−/−^ groups.

### 4.6 Geometric morphometric analysis

Geometric morphometric analysis was used to capture morphological shape variation between p75^NTR+/+^ and p75^NTR−/−^ mice. This method utilizes landmark-based data, allowing for more detailed analysis of shape differences that traditional linear measurements may overlook (Hallgrimsson et al., 2015; Hassan et al., 2020).

First, the Micro-CT data was imported into the 3D slicer software (slicer.org, Massachusetts, United States), and the cranium was manually segmented using the threshold parameters: 1,471 to 9800 HU. From these segmentations, isosurfaces were generated, and 41 landmarks were placed (Table 1). The landmark data was imported into the MorphoJ (ver. 2.0, Apache License, Klingenberg, C.P. 2011) program, and shape coordinate data were generated from the raw data using a Procrustes superimposition. Generalized Procrustes Analysis (GPA) was used to remove the effect of orientations, align configurations to a common centroid, and eliminate scale differences between the different samples. Principal component analysis (PCA) was used to identify shape differences and confirm the distinction between p75^NTR+/+^ and p75^NTR−/−^ mice. Outlier data points with outstanding distances from the cluster centers were removed.

### 4.7 Statistical analysis

We analyzed Procrustes shape data using multivariate analysis of variance (MANOVA). For the bivariate data sets, we illustrated data as means with their corresponding standard deviation. We then evaluated our conventional length and volumetric data by unpaired Student’s t-test. A p-value smaller or equal to 0.05 was used to indicate statistical significance.

## Data Availability

The original contributions presented in the study are included in the article/supplementary material, further inquiries can be directed to the corresponding author.
